# ELF5 drives angiogenesis suppression though stabilizing WDTC1 in renal cell carcinoma

**DOI:** 10.1186/s12943-023-01871-2

**Published:** 2023-11-18

**Authors:** Tushuai Li, Longjiang Xu, Zhe Wei, Shaomei Zhang, Xingyu Liu, Yanzi Yang, Yue Gu, Jie Zhang

**Affiliations:** 1 School of Biology and Food Engineering, Changshu Institute of Technology, 99 Southern Sanhuan Road, Suzhou, 215500 China; 2 Wuxi School of Medicine, Jiangnan University, Wuxi, 214013 China; 3 Department of Pathology, The Second Affiliated Hospital of Soochow University, Suzhou, 215000 China; 4 School of Pharmaceutical Sciences, Zhejiang Chinese Medical University, Hangzhou, 310053 China; 5 Key Laboratory of Anti-inflammatory and Immune Medicine, Ministry of Education, Institute of Clinical Pharmacology, Anhui Medical University, 81 Meishan Road, Hefei, 230032 China

**Keywords:** Renal cell carcinoma, ELF5, WDTC1, USP3, Angiogenesis, DNA methylation

## Abstract

**Background:**

Renal cell carcinoma (RCC) is a common malignant tumor of the urinary system. Angiogenesis is a main contributing factor for tumorigenesis. E74-like transcription factor 5 (ELF5) has been verified to participate in the progression of different cancers and can regulate angiogenesis. This study was aimed to explore the functions of ELF5 in RCC.

**Methods:**

Bioinformatics tools were used to predict the expression of ELF5 in RCC. RT-qPCR was applied for testing ELF5 expression in RCC cells. Cell behaviors were evaluated by colony formation, CCK-8, and transwell assays. The tube formation assay was used for determining angiogenesis. Methylation-specific PCR (MSP) was utilized for measuring the methylation level of ELF5 in RCC cells. ChIP and luciferase reporter assays were applied for assessing the binding of ELF5 and ubiquitin-specific protease 3 (USP3). Co-IP and GST pull-down were utilized for detecting the interaction of WD40 and tetratricopeptide repeats 1 (WDTC1) and USP3. Ubiquitination level of WDTC1 was determined by ubiquitination assay.

**Results:**

ELF5 was lowly expressed in RCC cells and tissues. High expression of ELF5 expression notably suppressed RCC cell proliferative, migratory, and invasive capabilities, and inhibited angiogenesis. The tumor growth in mice was inhibited by ELF5 overexpression. ELF5 was highly methylated in RCC samples, and DNA methyltransferases (DNMTs) can promote hypermethylation level of ELF5 in RCC cells. ELF5 was further proved to transcriptionally activate USP3 in RCC. Moreover, USP3 inhibited WDTC1 ubiquitination. ELF5 can promote USP3-mediated WDTC1 stabilization. Additionally, WDTC1 silencing reversed the functions of ELF5 overexpression on RCC progression.

**Conclusion:**

Downregulation of ELF5 due to DNA hypermethylation inhibits RCC development though the USP3/WDTC1axis in RCC.

**Supplementary Information:**

The online version contains supplementary material available at 10.1186/s12943-023-01871-2.

## Introduction

Renal cell carcinoma (RCC) is a common malignant tumor originating from the renal tubules [1]. Its incidence rate is only second to prostate cancer and bladder cancer, and it has been on the rise in the past decade [2]. Due to the lack of specific symptoms, RCC is usually diagnosed at a late stage, thus making it more difficult to treat [3]. The proportion of metastatic RCC patients is relatively high, resulting in a low survival of only about 13% [3]. At present, radical surgery remains the primary treatment for patients with early-stage RCC. Since RCC is not sensitive to radiotherapy, gene targeted therapy offers a promising new direction for its treatment [4]. Therefore, the identification of novel and feasible biomarkers is the key to developing better treatments and improving the survival of RCC patients.

Angiogenesis refers to the formation of new blood vessels from pre-existing ones, which is a crucial physiological process for tissue repair and development [5]. Accumulating studies suggest that angiogenesis is a key process in the development of solid tumors [6, 7]. It provides tumors with the oxygen and nutrients they need to grow, while allowing for the excretion of carbon dioxide and metabolites [8]. Furthermore, angiogenesis supports the metastasis of malignant cells from the primary tumor site to distant organs. Vascular endothelial growth factor (VEGF) is the most effective pro-angiogenic factor due to the mitogenic function [9]. In RCC, it has been reported that TNF receptor associated factor 1 can promote sunitinib resistance by enhancing angiogenesis [10]. F-Box Protein 22 inhibits tumor metastasis in RCC by suppressing cell migration and invasion and VEGF-mediated angiogenesis [11]. Silencing epidermal growth factor-like domain-containing protein 7 can suppress tumor growth by the inhibition of angiogenesis in RCC [12]. Therefore, the regulation on angiogenesis is crucial for developing new RCC treatment strategies.

E74-like transcription factor 5 (ELF5) belongs to the E26 transformation-specific (ETS) family which can modulate genes involved in cell proliferation, differentiation, apoptosis, and metastasis in human cancers [13]. Furthermore, ETS family have been confirmed to exert the crucial function in embryonic vasculogenesis, angiogenesis, and hematopoiesis [14, 15]. ELF5 is mainly expressed in epithelial cells and function as a suppressor of EMT of cancer cells [16]. Most evidence suggests that ELF5 exerts the vital function in inhibiting tumorigenesis and development in different human cancers. For example, ELF5 depletion can facilitate the metastasis of breast cancer by interferon-γ signaling [17]. ELF5 inhibits the epithelial-mesenchymal transition process in prostate cancer via inactivating SMAD3 [16]. ELF5 suppresses cell migration and invasion in ovarian cancer [18]. Importantly, ELF5 mRNA and protein have been confirmed to be decreased in RCC, and re-expression of ELF5 can suppress cell proliferation and survival, which indicates that ELF5 has tumor suppressive activity in the kidney [19]. However, the role of ELF5 in the angiogenesis of RCC is unknown.

Epigenetic alterations are the vital contributors to cancer development [20]. DNA methylation is the most common form of epigenetics, which can alter gene expression without altering DNA sequence [21]. Accumulating researches have confirmed that hypermethylation of gene promoter regions can directly lead to the decrease of gene expression levels [22]. DNA methylation is established by DNA methyltransferases (DNMTs) [23]. The 5-Aza-2-deoxycytidine (5-Aza-dC) is an inhibitor of DNMTs which causes genome-wide hypomethylation and inhibits cancer cell growth [24]. It has been reported that ELF5 downregulation in urothelial cancers is associated with DNA methylation, and it can be reversed by 5-Aza-dC [25]. Thus, DNA methylation may also explain the role of low-expressed ELF5 in the regulation of tumor activity in RCC.

This study aimed to investigate the function and mechanism of ELF5 in RCC. We evaluated ELF5 function on RCC cell behaviors through a series of functional assays. Mechanistically, we investigated the methylation status of ELF5 in RCC cells and the specific regulatory relationships between ELF5 and target genes.

## Materials and methods

### Clinical specimens

Tumor tissues and paracancerous tissues were obtained from 21 RCC patients received surgery in the Wuxi School of Medicine, Jiangnan University from 2020 to 2022. The detailed clinical information of these 21 patients (mean age 58.5 years; range 34–72 years) was listed in Table 1. The samples were quickly frozen in liquid nitrogen after surgery and then stored at the Wuxi School of Medicine until use. All samples were diagnosed by three pathologists independently. Patients didn’t receive preoperative radiotherapy, chemotherapy, targeted therapy, and immunotherapy prior to surgical excision. All procedures were approved by Ethics Committee of Wuxi School of Medicine, Jiangnan University. Patients had signed written informed consents.

**Table 1:** Clinicopathologic features of twenty-one patients

Patient serial number	Age	Gender	Tumor stage	Tumor grade	Histological subtype
1	76	Female	IV	3	pRCC I
2	67	Female	III	2	KIRC
3	56	Male	II	2	KIRC
4	45	Male	II	2	KIRC
5	33	Female	I	1	pRCC I
6	77	Male	II	1	chRCC
7	71	Male	IV	3	pRCC II
8	54	Male	II	3	KIRC
9	64	Female	II	2	KIRC
10	69	Male	IV	3	KIRC
11	64	Male	II	1	KIRC
12	47	Male	I	1	KIRC
13	48	Male	II	3	chRCC
14	70	Male	III	2	KIRC
15	60	Male	III	4	KIRC
16	56	Male	IV	4	KIRC
17	54	Female	II	2	KIRC
18	63	Female	IV	3	KIRC
19	52	Male	II	2	pRCC II
20	68	Female	III	3	KIRC
21	62	Female	III	3	chRCC

Note: Tumor stage was determined by the Robson system. For KIRC and pRCC, the four-tiered Fuhrman nuclear grading system was used. For chRCC, the three-tiered Panner grading system was sued. KIRC: kidney renal clear cell carcinoma; pRCC: papillary renal cell carcinoma; chRCC: chromophobe renal cell carcinoma

### Cell culture

The human RCC cell lines 769-P and A498, the normal human kidney 2 (HK-2) cells, and HUVEC were obtained from ATCC (Manassas, VA, USA). HUVECs were incubated in DMEM (Gibco, Carlsbad, CA) with 10% FBS. HK-2, 769-P, and A498 cells were incubated in RPMI-1640 medium (Gibco) containing 10% FBS. Cells were cultured at 37 °C with 5% CO_2_.

### Cell transfection

To silence DNMT1, DNMT3A, DNMT3B, or WDTC1 expression, the sh-DNMT1, sh-DNMT3A, sh-DNMT3B, or sh-WDTC1, as well as the negative control (sh-NC) purchased from GenePharma (Shanghai, China) were transfected into cells utilizing Lipofectamine 3000 (Invitrogen, Carlsbad, CA, USA) for 48 h. Further, the full-length sequence of ELF5, DNMT1, DNMT3B, WDTC1, or USP3 was inserted into pcDNA3.1 vector (Geenseed Biotech, Guangzhou, China), with the empty vector served as NC.

### RT-qPCR

TRIzol Reagent (Invitrogen) was utilized for the extraction of total RNA from cells. Then RNA was subjected to the reverse transcription to generate cDNA by the Reverse Transcription Kit (Qiagen, Hilden, Germany). The qPCR was carried out by Green Premix Ex Taq II (TaKaRa, Japan) on Step One Plus Real-Time PCR System (Applied Biosystems, Foster City, CA, USA). Gene expression was calculated by the 2^−ΔΔCt^ method normalized to GAPDH.

### Western blot

Total proteins were subjected to extraction from cells by RIPA buffer (Beyotime, China). Protein was isolated on a 12% SDS-PAGE and then transferred to polyvinylidene fluoride membranes. After blockading with 5% nonfat milk, membranes were probed with primary antibodies of VEGFA, ELF5, USP3, or WDTC1 (Abcam, USA) as well as the control GAPDH (Abcam) for one night at 4 °C, followed by culturing with secondary antibody (Abcam). Protein band was tested by the ECL kit (Millipore) and analyzed by ImageJ (v1.8.0; National Institutes of Health).

### CCK-8 assay

Cells were seeded into 96-well plate and cultured for 48 h. Then, 10 µL CCK-8 reagents (Dojindo, Kumamoto, Japan) was supplemented into each well for the further incubation of 2 h. The absorbance at 450 nm were tested through a microplate reader (Molecular Devices, USA).

### Colony formation assay

Cells were re-seeded in 6-well plates and incubated for fortnight. Cells were then fixed with methanol and dyed with 0.1% crystal violet. A microscope (Olympus, Japan) was applied for observing and counting their quantity.

### Transwell assay

The transwell chamber coating Matrigel (Corning) was applied for transwell invasion assay, and the migration assay was performed without Matrigel. Cells resuspended in 200 µL serum-free medium were put in the upper chamber, and 400 µL complete medium was implemented into the lower chamber. After 24 h, cells moved into the lower chamber were fixed and dyed by methanol and crystal violet. An inverted microscope (Olympus, Japan) was applied for observing and counting their quantity.

### Enzyme linked immunosorbent assay (ELISA)

VEGF content in cell supernatant was determined via the commercial ELISA Kit (R&D Systems, USA) in accordance with user guides. The absorbance value was tested through the microplate reader (Molecular Devices).

### Conditioned medium for HUVECs

A498 and 769-P cells (8 × 10^5^) were seeded in a 6-cm culture dish for adhesion. Then cells were rinsed thrice with serum-free medium (SFM), treated with 4 mL of SFM, and incubated for 24 h. After centrifugation, the supernatant was gathered as conditioned medium (CM) for HUVECs.

### Tube formation assay

HUVECs mixed with CM or SFM were put in a 24-well plate with Matrigel for 4 h. Then, the inverted microscope (Olympus) was employed for observing the tube-like vascular structures.

### Xenograft models

Animal assays were approved by the Ethics Committee of Wuxi School of Medicine, Jiangnan University. Six-week-old male BALB/C nude mice were obtained from Wuxi School of Medicine, Jiangnan University. Stable transfection of control vector (pcDNA3.1), pcDNA3.1-ELF5 and ELF5-ΔSET was performed in the 769-P cell line, which was subsequently used for animal studies. To explore the effects of 5-Aza-dC on tumor growth in vivo, each mouse was intraperitoneally administrated with 30 mg/kg 5-Aza-dC or same dose of PBS daily for a week. The mice were euthanized after 40 days, and tumor volume and weight were detected. The tumors in mice were visualized by bioluminescence imaging utilizing IVIS Lumina II system (Caliper Life Sciences, Hopkinton, MA, USA).

### Immunohistochemistry (IHC) assay

The paraffin-embedded sections were dewaxed and rehydrated. Antigen retrieval was conducted with Target Retrieval Solution (Dako, CA, USA). Endogenous peroxidase was blocked by 3% H_2_O_2_. After blockading nonspecific antigen binding with 5% BSA, sections were cultured with the primary antibody against ELF5, Ki67 or VEGFA (Abcam, USA) at 4 °C for one night. Then sections were cultured with the secondary antibodies (Abcam, USA) at 37 °C for one hour. Later, they were dyed with DAB and counterstained with hematoxylin. The light microscope was applied for observation. In accordance with the method described by Weidner et al. [26], we evaluated the microvessel density (MVD). The quantity of CD34-positive cells was recorded as the value of MVD.

### Methylation-specific PCR (MSP)

Genomic DNA was subjected to extraction from cells by the QIAamp DNA Mini Kit (Qiagen). After purification, the DNA was exposed to bisulfite with the EpiTect Bisulfite Kit (Qiagen) in accordance with user guides. Utilizing the GeneAmp PCR System 2700 (Applied Biosystems), the MSP of bisulfite-transformed DNA was implemented.

### Chromatin immunoprecipitation (ChIP) assay

A ChIP assay kit (Beyotime) was employed for this assay. Cells were cross-linked with formaldehyde and sonicated to an average length of 200–1000 bp. Cell lysates were cultured with protein A/G beads coated with the antibody against ELF5, ETS1, SPI1, or IgG (Abcam, USA). DNA Extraction Kit (GeneMark) was utilized for purifying the cross-linked DNA. After elution, DNA was analyzed by RT-qPCR.

### Luciferase reporter assay

USP3 promoter region containing ELF5 putative binding sites (wild type, WT) or mutant (mutant type, MUT) was cloned into the pGL3-basic vector. Cells were subjected to co-transfection with the luciferase vectors and indicated plasmids for 48 h. After that, a Dual-Luciferase Reporter Assay System (Promega, USA) was applied for measuring the luciferase activity.

### Co-immunoprecipitation (Co-IP)

The Pierce Classic Magnetic Co-IP Kit (Thermo, USA) was applied for this assay. Cells were treated with IP lysis buffer for obtaining the supernatant. Cell lysates were cultured with antibodies against USP3 or WDTC1 at 4 °C for one night. Then the immunoprecipitation complex was formed. Later, the protein A/G beads were supplemented and cultured for 1 h. After that, the supernatant was used for western blot analysis.

### GST pull-down assay

After IPTG induction, the encoded USP3-GST fusion and the control GST proteins were expressed in BL21 cells. The GST-fusion proteins were arrested by glutathione sepharose 4B beads (GE Healthcare, UK) for the GST pulldown assays, and Flag-WDTC1 was supplemented. After three hours for combination at the temperature of 4 °C, beads were rinsed by PBS and boiled in SDS loading buffer. The bound proteins were loaded onto a 12% SDS-PAGE, followed by analysis of western blot.

### Ubiquitination analysis

The whole-cell lysates (WCL) were prepared with the RIPA buffer added with N-Ethylmaleimide and protease inhibitor. After boiling at 95 °C for 15 min, the lysate was treated with 0.1% SDS comprising RIPA buffer. Then, centrifugation was performed, and the supernatant was cultured with antibodies together with protein A-Sepharose beads for one night at 4 °C. The beads were boiled with loading buffer and analyzed by immunoblotting.

### Statistical analyses

GraphPad Prism 8 software was employed for statistical analysis. Data were expressed as the means ± standard deviation (SD) from three individual repeats. Data were analyzed by Student’s *t*-test or one-way analysis of variance. *p* < 0.05 was considered significant.

## Results

### ELF5 is the potential tumor suppressor gene in RCC

**Fig. 1:**
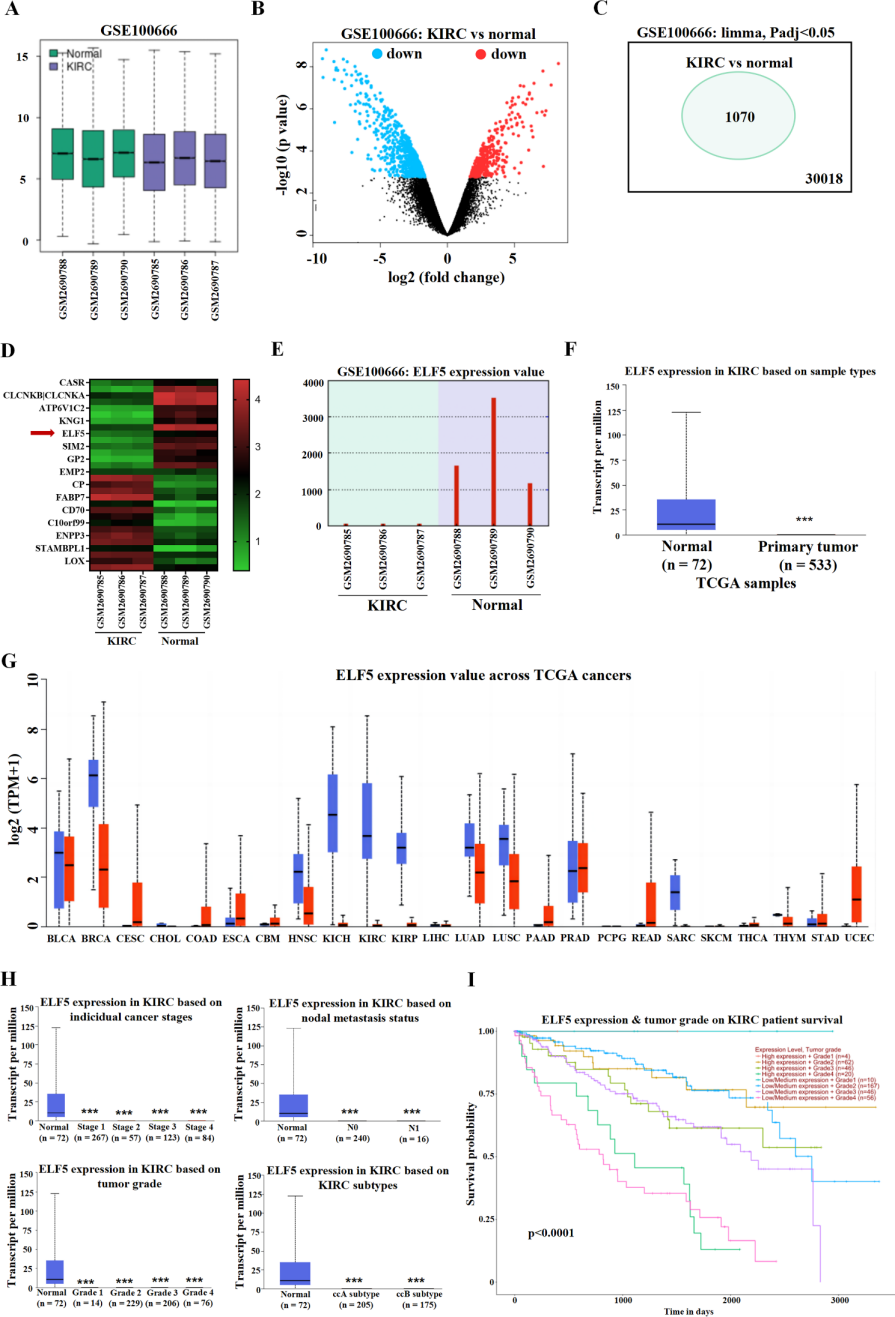
ELF5 is the potential tumor suppressor gene in RCC. **(A)** GSE100666 dataset showed the KIRC samples (GSM2690785, GSM2690786, and GSM2690787) and paracancerous tissue samples (GSM2690788, GSM2690789, and GSM2690790). The ordinate represented the overall expression level of all samples **(B)** The volcano map of GSE100666. **(C)** One thousand and seventy differentially expressed genes were screened out. **(D)** The heat map of genes. **(E)** GSE100666 dataset showed ELF5 expression in KIRC and non-tumoral tissues, and the ordinate represented the expression level of ELF5 in 6 samples. **(F)** UALCAN database showed ELF5 expression in TCGA-KIRC and non-tumoral tissues. **(G)** The pan-cancer analysis showed ELF5 expression in different cancers. **(H)** ELF5 expression in the cancer stages, tumor grades, nodal metastasis status, and KIRC subtypes. **(I)** The survival analysis of patients with high/low ELF5 and different tumor grades. Two-tailed Student’s t test was performed. The error bars represent SD. ***p < 0.001

Through the GSE100666 dataset, we explored the differentially expressed genes in RCC tissues and paraneoplastic tissues. Among them, GSM2690785, GSM2690786, and GSM2690787 were kidney renal clear cell carcinoma (KIRC) tissue samples, and GSM2690788, GSM2690789, and GSM2690790 were paraneoplastic non-tumoral samples (Fig. 1A). The general consistency of the sample size can provide a guarantee of the reliability of the data analysis results. Then we screened the differentially expressed genes and obtained a total of 30,018 differentially expressed genes, of which 1070 genes with significant differences (Fig. 1B-C). As shown in Fig. 1D, the heat map showed the top 30 most significantly and differentially expressed genes with high or low expression, among which ELF5 caught our attention. Based on the cBioPortal platform, we found that ELF5 expression was accompanied by gene alterations in multiple cancers such as uterine endometrioid carcinoma, breast cancer and colorectal cancer, but showed no alterations in renal cell carcinoma (Fig. S1A-B). The expression pattern of members of the ETS transcription factor family was shown in Fig. S1C, and UALCAN (https://ualcan.path.uab.edu/index.html) database showed that ELF5 was downregulated in kidney renal clear cell carcinoma (KIRC) (Fig. 1F). Further, by GSE100666 analysis, we also found that ELF5 was abnormally lowly expressed in KIRC tissues (Fig. 1E). Similarly, ELF5 was significantly low expressed in KIRC tissues (Fig. 1F). Pan-cancer analysis indicated that ELF5 expression was downregulated in most cancers and was an extensive cancer suppressive factor potentially (Fig. 1G). In addition, in KIRC samples, we found that ELF5 was significantly low expressed in cancer stages, tumor grades, lymph node metastasis and subtypes (Fig. 1H). Survival analysis further showed that, under the same tumor grade, high expression of ELF5 predicted better prognosis, while low expression of ELF5 had poorer prognosis (Fig. 1I). Therefore, we speculated that ELF5 function as a potential oncogene in RCC.

### High ELF5 expression inhibits RCC cell proliferation, migration, and invasion

**Fig. 2:**
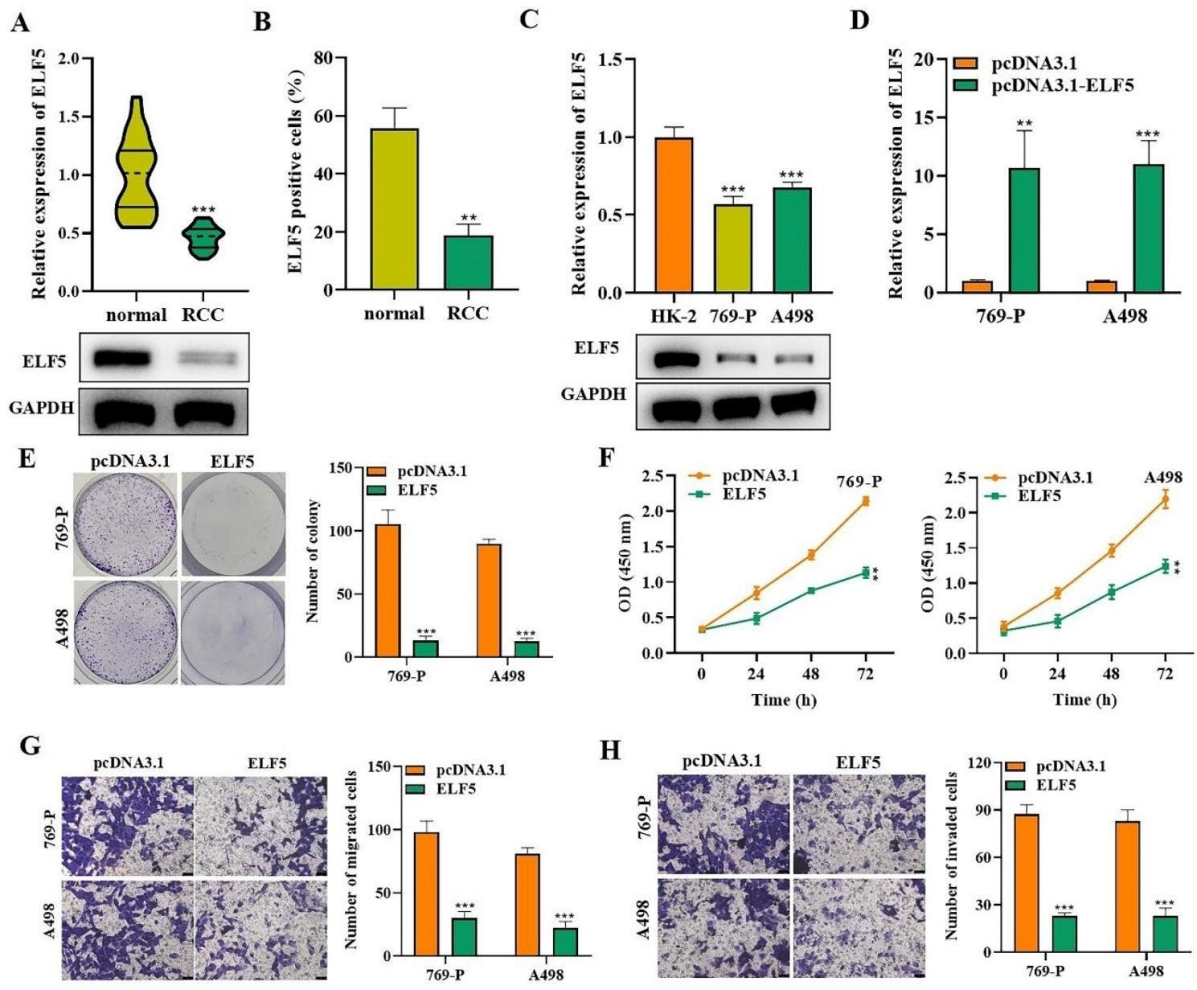
High ELF5 expression inhibits RCC cell proliferation, migration, and invasion. **(A)** RT-qPCR and western blot outcomes of ELF5 expression in30 paired RCC tissues (T) and adjacent normal tissues (N). **(B)** IHC staining assay was utilized for detecting the staining intensity of ELF5 in RCC tissues. **(C)** RT-qPCR and western blot outcomes of ELF5 levels in HK-2, 769-P, and A498 cells. **(D)** Western blot outcomes of the expression of ELF5. **(E-F)** Colony formation and CCK-8 assays were implemented for detecting proliferative capability when ELF5 / ELF5-ΔSET was overexpressed in cells. **(G-H)** Transwell assay was carried out for testing cell migratory and invasive capabilities. Two-tailed Student’s t test was performed. The error bars represent SD. **p < 0.01, and ***p < 0.001

We further assessed ELF5 expression in RCC and discovered ELF5 expressed at a low level in tumor tissues of RCC patients compared to non-tumoral tissues (Fig. 2A). IHC assay further verified this finding (Fig. 2B). Next, in RCC cell lines 769-P and A498, we observed the significant decrease of ELF5 expression (Fig. 2C). To explore the individual role of EZH2 function, we made 769-P and A498 stable cells either overexpressing ELF5 or overexpressing ELF5 transcriptional inactive (–ΔSET). Western blot results verified the efficiency of ELF5 overexpression (Fig. 2D). By colony formation assay and CCK-8 assay, we discovered ELF5 upregulation markedly reduced the quantity of colonies and OD value, indicating that cell proliferation was effectively inhibited, while ELF5-ΔSET had no significant change in RCC cell proliferation (Fig. 2E-F). Similarly, the transwell assays further manifested cell migratory and invasive abilities were diminished via ELF5 but not ELF5-ΔSET overexpression (Fig. 2G-H). Therefore, we concluded that ELF5 overexpression could suppress RCC cell behaviors.

### ELF5 acts as an angiogenesis inhibitor in RCC

**Fig. 3:**
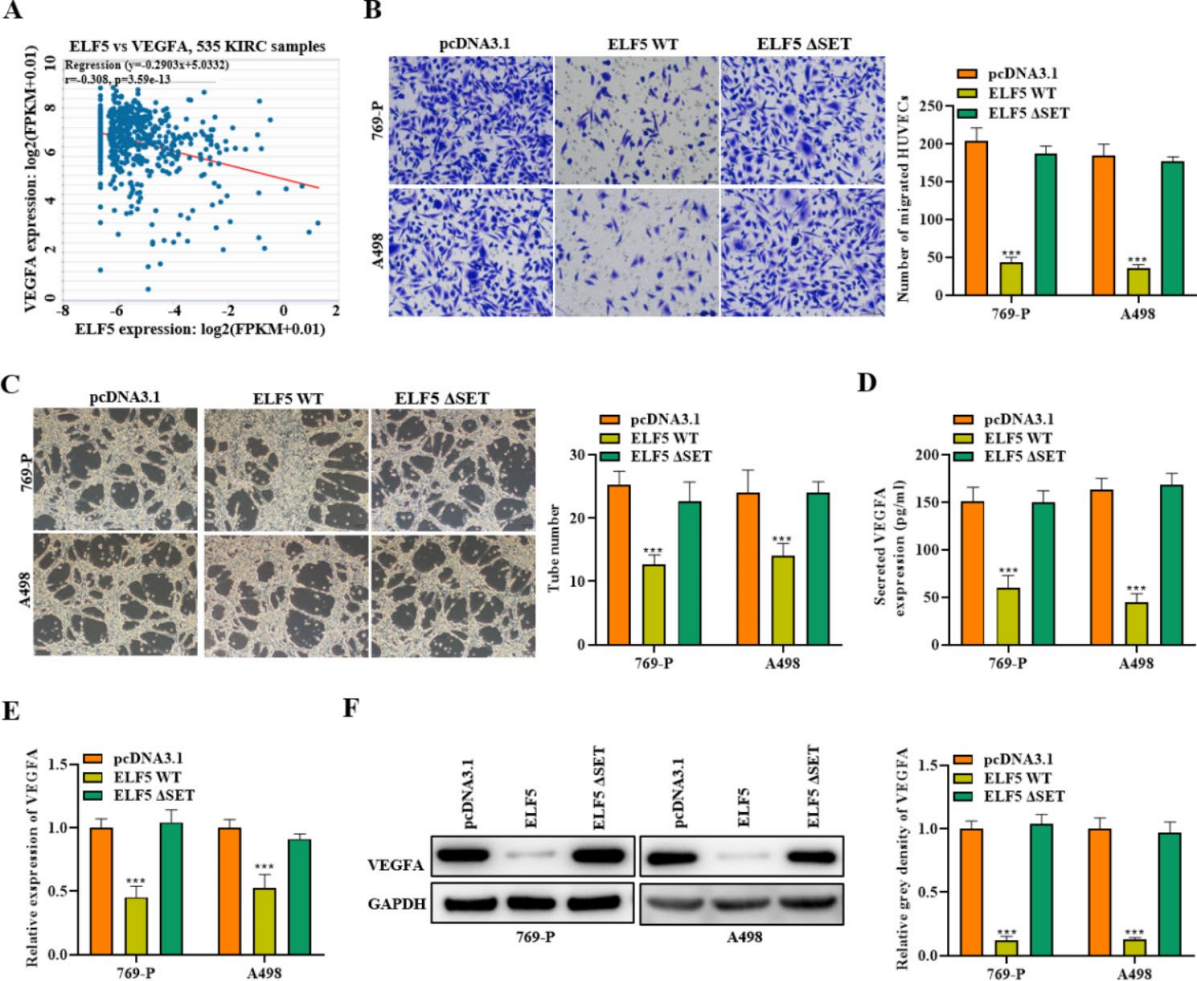
ELF5 acts as an angiogenesis inhibitor in RCC. **(A)** ENCORI database showed the correlation analysis of ELF5 and VEGFA expression. **(B)** Cell migration was determined by transwell assay after overexpressing ELF5 / ELF5-ΔSET. **(C)** Tube formation assay was employed for testing angiogenesis of HUVECs stimulated by the conditioned medium collected from the transfected 769-P and A498 cells. **(D)** ELISA results of VEGFA concentration in the conditioned medium of transfected 769-P and A498 cells. **(E-F)** RT-qPCR and western blot outcomes of VEGFA level in different cells. Two-tailed Student’s t test was performed. The error bars represent SD. ***p < 0.001

ELF5 and its ETS family have been confirmed to regulate angiogenesis in cancers [18], so we conducted assays to determine whether ELF5 regulates angiogenesis in RCC. Correlation analysis by the ENCORI database revealed a significant negative correlation between ELF5 and vascular endothelial growth factor A (VEGFA) expression, suggesting a repressive role of ELF5 in angiogenesis (Fig. 3A). Next, we collected culture medium from 769-P and A498 cells to stimulate HUVECs and tested the function of ELF5 upregulation on cell behaviors. Transwell assay manifested the migration of ELF5-overexpressed cells was obviously reduced compared to control cells (Fig. 3B). Tube formation assay demonstrated that ELF5 upregulation notably weakened the angiogenesis ability of HUVECs (Fig. 3C). Further, we examined the expression alteration of VEGFA in HUVECs. ELISA showed that VEGFA concentration was notably reduced by ELF5 overexpression in the conditioned medium of transfected 769-P and A498 cells (Fig. 3D). Moreover, we discovered VEGFA expression levels were reduced in ELF5-overexpressed cells in comparison of control cells (Fig. 3E-F). Nevertheless, overexpression of ELF5–ΔSET remained unchanged as compared to control and exhibited no change in angiogenesis of RCC. These results confirmed that ELF5 inhibited HUVEC migration and angiogenesis, suggesting that ELF5 acted as an angiogenesis suppressor in RCC and ETS domain of ELF5 is responsible of its angiogenesis suppressor activities upon ectopic expression RCC cell lines.

### ELF5 inhibits tumor growth and angiogenesis in RCC

**Fig. 4:**
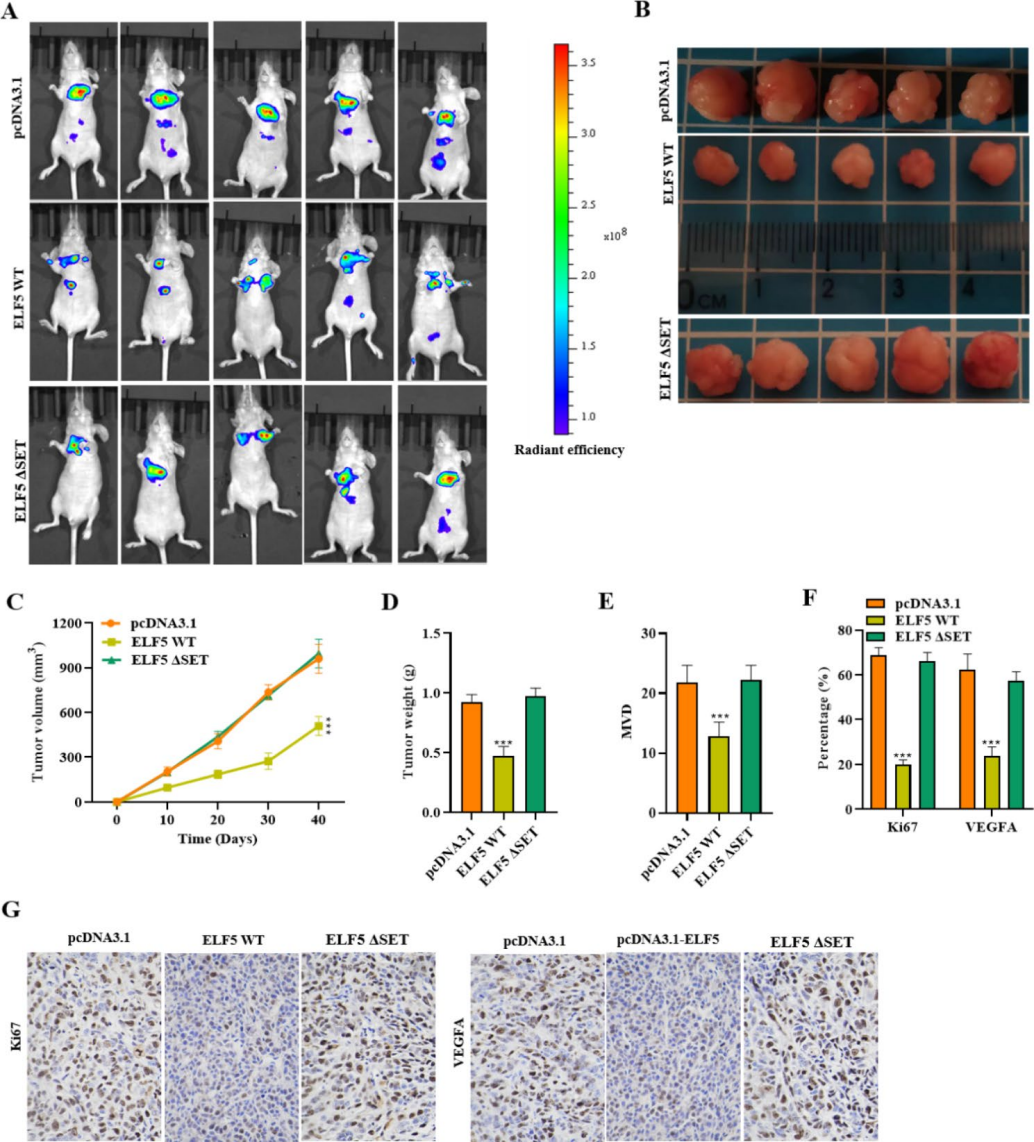
ELF5 suppresses RCC tumor growth. **(A)** Real-time imaging system was applied for detecting the luminescent images of tumors of mice in the pcDNA3.1 group, the pcDNA3.1-ELF5 group and pcDNA3.1-ELF5-ΔSET. **(B-D)** Morphology of the tumors, tumor volume, and weight of mice. **(E)** MVD in tumor tissues of the pcDNA3.1 group, the pcDNA3.1-ELF5 group and the pcDNA3.1-ELF5-ΔSET group was determined. **(F-G)** IHC results of Ki67 and VEGFA expression in tumor tissues. Two-tailed Student’s t test was performed. The error bars represent SD. ***p < 0.001

The regulation of ELF5 in vitro encouraged us to explore further the impact of ELF5 on RCC tumor growth. The mouse model was established for further examining ELF5 function on tumor growth in vivo. A real-time imaging system was applied for detecting luminescent images of tumors, and the images showed significantly smaller tumors in mice injected with 769-P cells stably expressing ELF5 or ELF5-ΔSET (Fig. 4A). Figure 4B specifically shows the tumor morphology in the pcDNA3.1-NC and pcDNA3.1-ELF5 groups. The tumor volume and weight were also obviously lower in the ELF5-overexpressed mice, while the tumor volume and weight in the ELF-ΔSET treated group were not significantly different from those in the control group (Fig. 4C-D). Similarly, we further found that ELF5 upregulation notably suppressed MVD in tumor tissues, instead of ELF-ΔSET (Fig. 4E). IHC results manifested that Ki67 and VEGFA expression in tumor tissues was decreased via ELF5 but not ELF-ΔSET overexpression (Fig. 4F-G). Collectively, ELF5 overexpression repressed tumor growth and angiogenesis in vivo. Altogether, overexpression of ELF5-ΔSET remained unchanged as compared to control and exhibited no change in tumor growth and angiogenesis of RCC whereas WT ELF5 overexpression resulted in decreased tumor progression suggesting the crucial role of ELF5 in suppressing RCC tumor.

### DNMTs promote hypermethylation of ELF5 in RCC

**Fig. 5:**
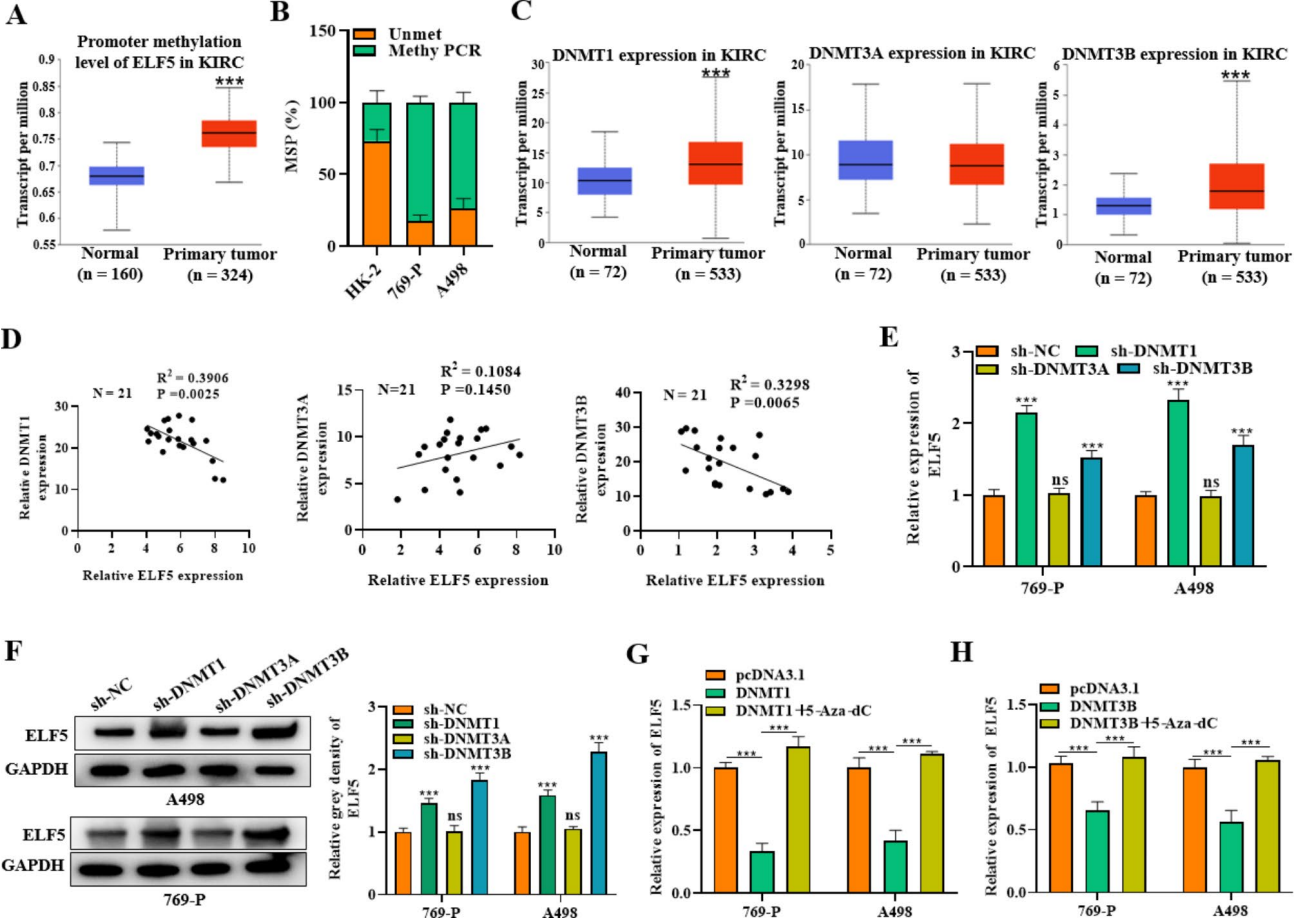
DNMTs promote hypermethylation of ELF5 in RCC. **(A)** UALCAN database was utilized to predict ELF5 methylation status in KIRC samples and normal samples. **(B)** MSP assay was utilized to detect ELF5 methylation in HK-2, 769-P, and A498. **(C)** UALCAN database was utilized for predicting DNMT1, DNMT3A, and DNMT3B expression in KIRC. **(D)** The correlation between ELF5 and DNMT1/DNMT3A/DNMT3B in RCC patient tissues (n = 21). **(E-F)** ELF5 expression was determined by RT-qPCR and western blot in cells when DNMT1, DNMT3A, or DNMT3B was silenced. **(G-H)** ELF5 level was determined through RT-qPCR when DNMT1/DNMT3B was overexpressed, and 5-Aza‐dC was added in cells. Two-tailed Student’s t test was performed. The error bars represent SD. ns means no significance, ^***^p < 0.001

The crucial function of epigenetic alterations in carcinogenesis and metastasis has received more and more attention [20]. DNA methylation is a crucial epigenetic modification of organisms, and is catalyzed by DNMTs (DNMT1, DNMT3A and DNMT3B). We further evaluated whether the low expression ELF5 in RCC was mediated by DNA hypermethylation [27]. Through analysis of the UALCAN database, we found that ELF5 was highly methylated in the tissues of KIRC (Fig. 5A). MSP assay was utilized to further examine ELF5 methylation in HK-2, 769-P and A498 cells. The methylation level of ELF5 was significantly elevated in RCC cells in comparison of normal cells (Fig. 5B). Next, UALCAN database showed that DNMT1 and DNMT3B were significantly highly expressed in KIRC tissues, and DNMT3A expression was not significantly different between KIRC tissues and non-tumoral tissues (Fig. 5C). The correlation between ELF5 and DNMTs was further analyzed in RCC patient tissues. We observed that ELF5 was negatively related to DNMT1 and DNMT3B, and insignificantly related to DNMT3A (Fig. 5D). Moreover, ELF5 expression was elevated by knockdown of DNMT1 or DNMT3B; however, DNMT3A depletion had almost no effect on ELF5 expression (Fig. 5E-F). Further, we observed that ELF5 expression was significantly decreased in cells overexpressing DNMT1 or DNMT3B compared with control cells, to which ELF5 expression was significantly rebounded by the addition of the methylation inhibitor 5-Aza-dC treatment (Fig. 5G-H). Moreover, the effects of 5-Aza-dC treatment on RCC tumor growth was further investigated. We found that the tumor size and weight was significantly reduced in the 5-Aza-dC treatment group relative to the control group (Fig. S2A-B). The MVD in mouse tumor tissues was also decreased by the administration with 5-Aza-dC (Fig. S2C). We also confirmed that the expression of ELF5 was upregulated in the mouse tumor tissues in the 5-Aza-dC group compared with the control group (Fig. S2D). Collectively, we confirmed that DNMTs promoted the hypermethylation of ELF5, leading to a downregulation of ELF5 levels in RCC cells. 5-Aza-dC as a DNA methyltransferase inhibitor showed the potential for RCC tumor suppression via ELF5.

### ELF5 transcriptionally activates USP3

**Fig. 6:**
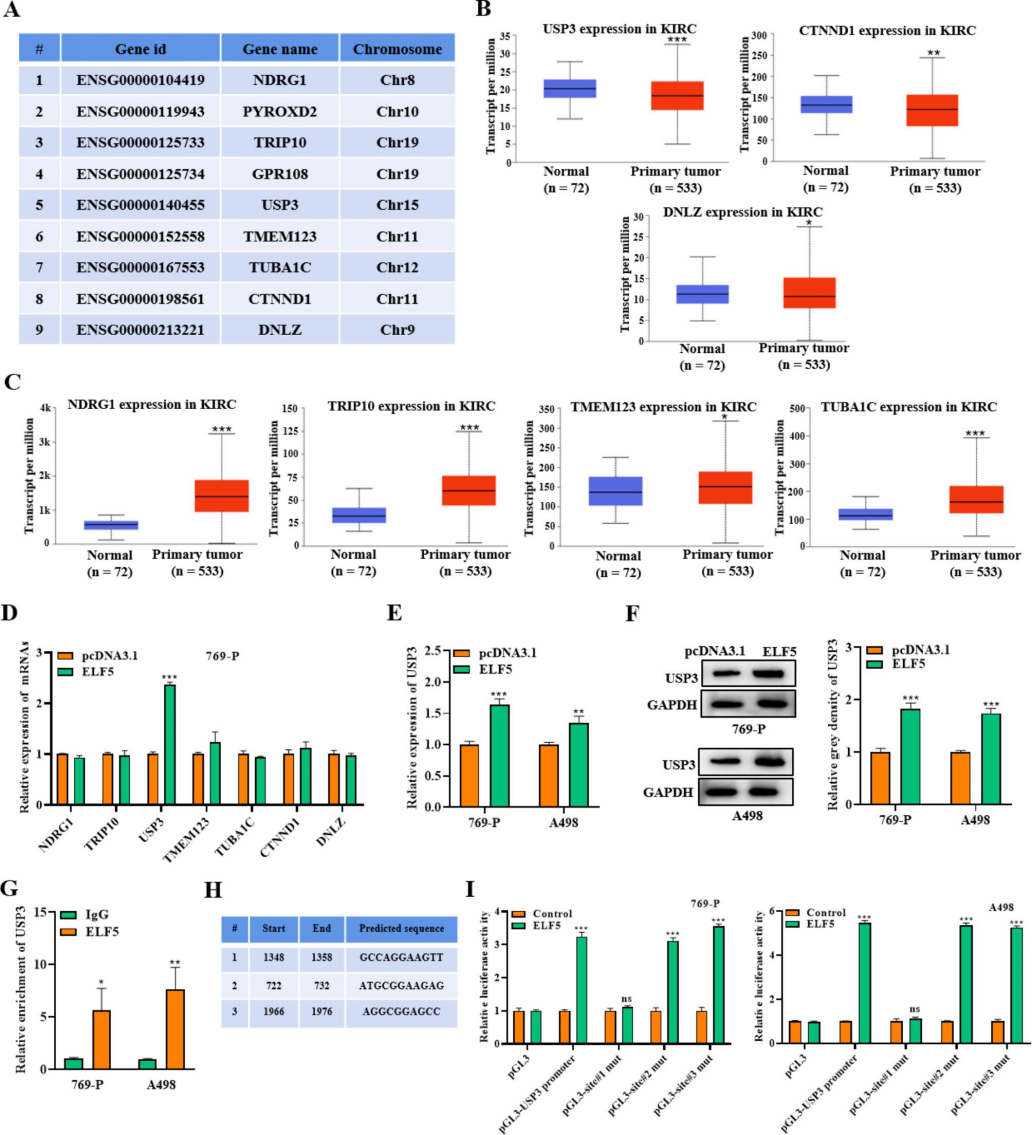
ELF5 transcriptionally activates USP3. **(A)** The hTFtarget database was employed for predicting target genes for ELF5. **(B-C)** UALCAN database was employed for predicting the expression of candidate target genes in KIRC. **(D)** The effect of ELF5 overexpression on the expression of the candidate genes was detected by RT-qPCR in 769-P cells. **(E-F)** RT-qPCR and western blot results of USP3 levels in cells transfected with pcDNA3.1-NC or pcDNA3.1-ELF5. **(G)** ChIP assay was utilized for determining the interaction of ELF5 and USP3 promoter. **(H)** The binding sites of ELF5 on USP3 promoter. **(I)** Luciferase reporter assay was carried out for validating the binding of ELF5 on USP3 promoter. Two-tailed Student’s t test was performed. The error bars represent SD. *p < 0.05, **p < 0.01, and ***p < 0.001

For further investigating the specific regulatory mechanism of ELF5 in RCC, the target genes of ELF5 were predicted utilizing the hTFtarget (http://bioinfo.life.hust.edu.cn/hTFtarget#!/) database (Fig. 6A). Then the UALCAN database was used to analyze the expression differences of candidate target genes in KIRC samples. The results illustrated that USP3, CTNND1, and DNLZ were markedly low expressed in KIRC (Fig. 6B). NDRG1, TRIP10, TMEM123, and TUBA1C were significantly high expressed in KIRC (Fig. 6C). The expression differences of PYROXD2 and GPR108 in KIRC and non-tumoral samples were not significant, thus they were excluded. Next, in 769-P cells, we determined the effect of ELF5 on the expression of these candidate target genes using RT-qPCR. As a result, only USP3 expression was notably upregulated by ELF5 overexpression (Fig. 6D). Next, we observed ELF5 overexpression could effectively enhance the mRNA and protein levels of USP3 in 769-P and A498 cells (Fig. 6E-F). Based on the hTFtarget database, two other ETS transcription factors (ETS1 and SPI1) were predicted to be the transcription factors for USP3 in kidney tissues. However, the results of ChIP assays indicated that USP3 was not significantly enriched in the anti-ETS1 and anti-SPI1 groups, suggesting that USP3 was not specifically targeted by ETS1 and SPI1 in RCC cells (Fig. S1D-E). Subsequently, ChIP assays illustrated that USP3 was abundantly enriched by anti-ELF5, validating the targeting of ELF5 with the USP3 promoter (Fig. 6G). By prediction of JASPAR (https://jaspar.genereg.net/) database, we obtained three binding sites for ELF5 to USP3 promoter (Fig. 6H). The luciferase reporter assay manifested that ELF5 overexpression obviously elevated the luciferase activity of the USP3 promoter. However, after mutating site#1, the luciferase activity was significantly reduced, indicating that site#1 was the site where ELF5 targeted the USP3 promoter (Fig. 6I). These results confirmed that ELF5 transcriptionally activated USP3 in RCC. Following, we measured USP3 function on the cell malignant phenotypes. RT-qPCR showed that USP3 expression was downregulated in RCC cells (Fig. S3A). We then overexpressed USP3 in the cells and performed gain-of-function assays. The results verified USP3 overexpression reduced colonies, migrated cells, and invaded cells, demonstrating USP3 could suppress RCC cell proliferation, migration, and invasion (Fig. S3B-D). Moreover, the tube formation assay illustrated that USP3 could inhibit angiogenesis (Fig. S3E). In conclusion, we authenticated that USP3 suppressed RCC progression and was regulated by ELF5.

### ELF5 promotes USP3-mediated WDTC1 stabilization

**Fig. 7:**
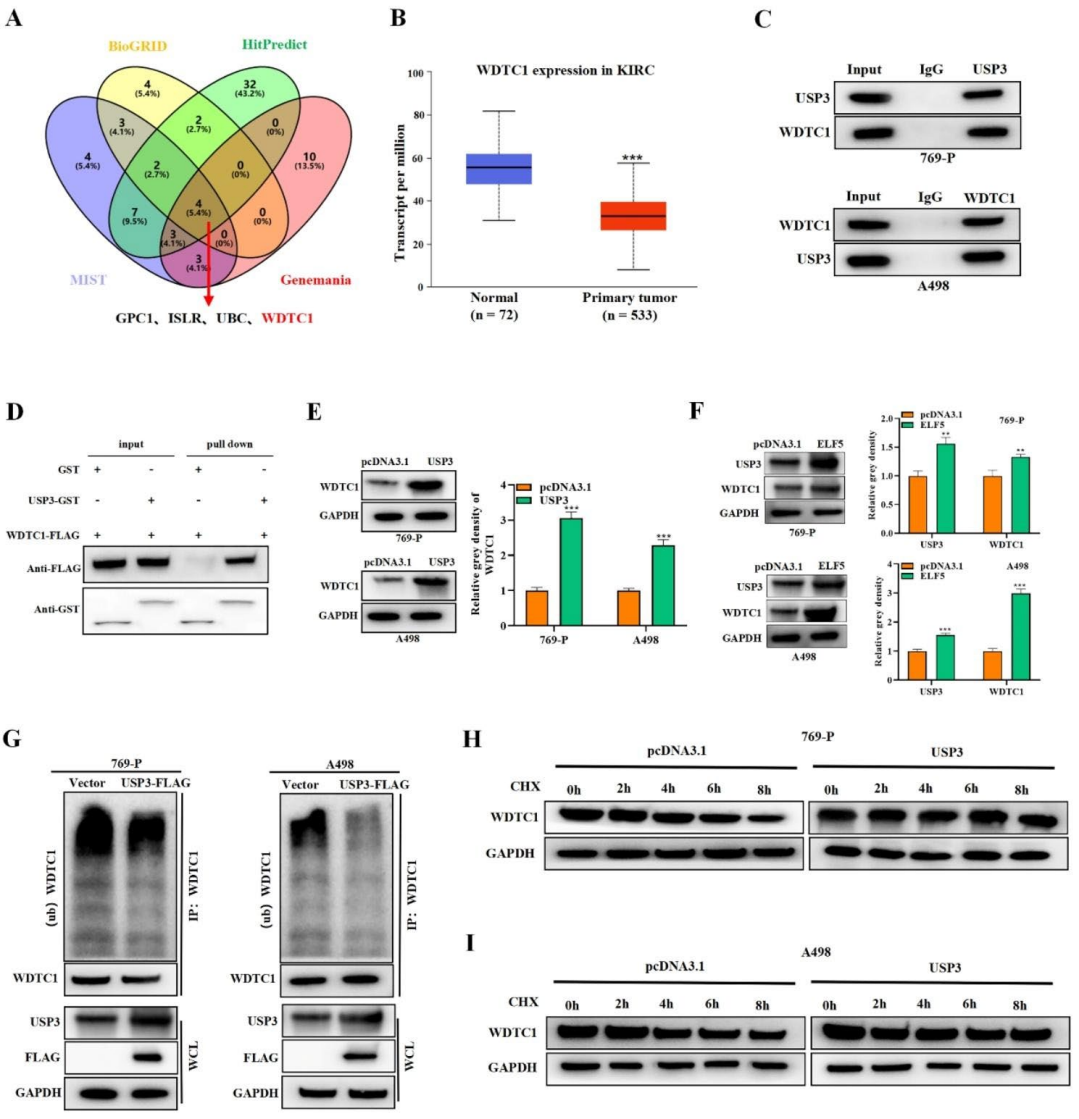
ELF5 enhances USP3-mediated WDTC1 stabilization. **(A)** BioGRID, HitPredict, MIST and Genemania database were employed for predicting the reciprocal protein of USP3. **(B)** UALCAN database was utilized to predict WDTC1 expression in KIRC. **(C)** Co-IP assay was implemented for detecting the endogenous interplay between USP3 and WDTC1. **(D)** GST pull-down assay was utilized to verify the direct interaction between USP3 and WDTC1. **(E)** Western blot results of WDTC1 level when USP3 was overexpressed in cells. **(F)** Western blot results of WDTC1 and USP3 levels in cells when ELF5 was overexpressed in cells. **(G)** Ubiquitination assay was implemented to test the impact of USP3 on WDTC1 ubiquitination level. **(H-I)** Western blot was implemented to determine WDTC1 level in CHX-treated cells when USP3 was overexpressed. Two-tailed Student’s t test was performed. The error bars represent SD. ***p < 0.001

We further used the BioGRID (https://thebiogrid.org/), HitPredict (http://www.hitpredict.org/), MIST (https://mistdb.com/) and Genemania (http://genemania.org/) databases jointly to predict the USP3 reciprocal proteins. The results displayed that GPC1, ISLR, UBC, and WDTC1 were potential reciprocal proteins for USP3 (Fig. 7A). UALCAN database was applied for analyzing the expression of candidate reciprocal proteins in KIRC samples. As a result, WDTC1 expression was markedly downregulated in KIRC, suggesting WDTC1 may be a reciprocal protein for USP3 (Fig. 7B). The results of Co-IP assay confirmed that USP3 and WDTC1 can endogenously interact with each other (Fig. 7C). GST pull-down assay further verified that USP3 and WDTC1 can directly interact with each other (Fig. 7D). We then observed that USP3 overexpression significantly upregulated WDTC1 protein levels in cells by western blot (Fig. 7E). Further, ELF5 overexpression was found to upregulate USP3 and WDTC1 levels (Fig. 7F). Next, ubiquitination assays showed that USP3 notably suppressed the ubiquitination level of WDTC1 in 769-P and A498 cells (Fig. 7G). Subsequently, we examined WDTC1 protein levels using western blot after treating cells with the protein synthesis inhibitor CHX (325 ng/ml). We found that the protein half-life of WDTC1 was longer in USP3-overexpressed cells in the presence of CHX, confirming that USP3 inhibited the ubiquitination degradation of WDTC1 in RCC cells (Fig. 7H-I). Therefore, we suggested that ELF5 promoted USP3 to suppress WDTC1 ubiquitination, thus improving the stability of WDTC1.

### ELF5 inhibits RCC progression by upregulating WDTC1

**Fig. 8:**
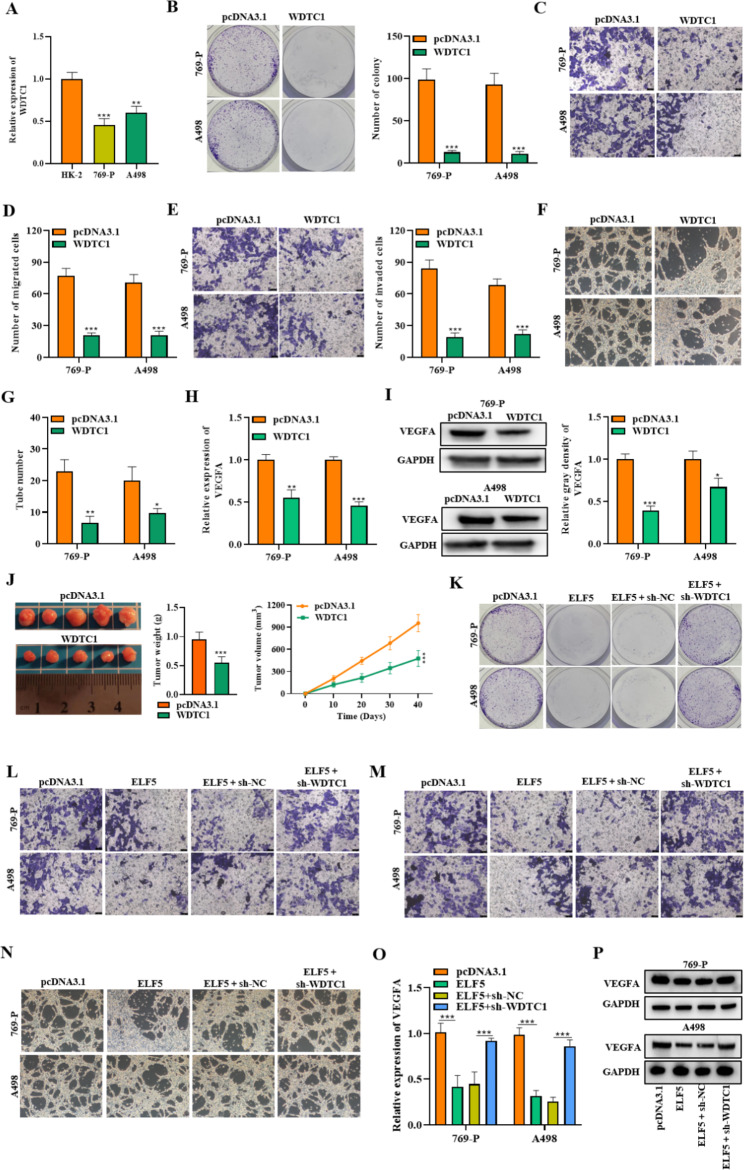
ELF5 inhibits RCC progression by upregulating WDTC1. **(A)** RT-qPCR result of WDTC1 expression in HK-2, 769-P, and A498 cells. **(B)** Colony formation assay was employed for detecting cell proliferative capability when WDTC1 was overexpressed. **(C-E)** Transwell assay was carried out for testing cell migratory and invasive capabilities. **(F-G)** Tube formation assay was employed for testing angiogenesis of HUVECs. **(H-I)** RT-qPCR and western blot outcomes of VEGFA level.**(J)** Tumor weight and the tumor growth curve were measured in WDTC1 overexpression cell and its control group **(K-P)** Cell proliferation **(k)**, migration **(l)**, invasion **(m)**, angiogenesis **(n)**, and VEGFA expression **(o-p)** were detected by colony formation assay (k), transwell assay **(l-m)**, tube formation assay (n), RT-qPCR (o), and western blot (p) in cells of the pcDNA3.1 group, the pcDNA3.1-ELF5 group, the pcDNA3.1-ELF5 + sh-NC group, and the pcDNA3.1-ELF5 + sh-WDTC1 group. Two-tailed Student’s t test was performed. The error bars represent SD. *p < 0.05, **p < 0.01, and ***p < 0.001

WDTC1 was found to be lowly expressed in RCC cells (Fig. 8A). Then colony formation assay manifested that WDTC1 overexpression effectively reduced cell proliferative capability (Fig. 8B). Through transwell assay, we proved that cell migration and invasion can be suppressed by upregulating WDTC1 (Fig. 8C-E). Additionally, tube formation assay manifested that WDTC1 overexpression notably inhibited angiogenesis ability (Fig. 8F-G). The expression of VEGFA in cells was also decreased by WDTC1 overexpression (Fig. 8H-I). In-vivo studies established that WDTC1 inhibited RCC tumor growth (Fig. 8J). These results proved that WDTC1 can function as a tumor suppressor in RCC. Next, we determined the impact of WDTC1 silencing on RCC cell behaviors. The outcomes verified that cell proliferation, migration, invasion, and angiogenesis suppressed by ELF5 overexpression were significantly reversed by WDTC1 silencing (Fig. 8K-P). Thus, we confirmed that ELF5inhibits RCC progression by upregulating WDTC1.

## Discussion

RCC is a common malignant tumor. Current treatment modalities are not effective in improving the survival rate of RCC patients. In recent years, gene targeted therapy has been proven in assorted cancers [28, 29]. CiRS-7 has been confirmed as the prognostic biomarker and potential gene therapy target for RCC [30]. ELF5 and its ETS family have been verified to modulate cell proliferation, differentiation, metastasis, and angiogenesis in tumors [17]. Several studies have demonstrated that ELF5 is lowly expressed in human cancers and acts as an oncogene regulator, including RCC [19, 31, 32]. Bioinformatics tools showed that ELF5 expressed at a low expression in tumor stages, tumor grades, lymph node metastasis, and subtypes of KIRC. Similarly, we found ELF5 expression was downregulated in RCC tissues and cells. ELF5 upregulation notably repressed RCC cell proliferation, migration, invasion, and tumor growth. Accumulating studies suggest that angiogenesis is a key process in the development of solid tumors [33]. VEGFA is a major factor driving the expansion of tumor vascular beds [34]. We further proved that ELF5 upregulation inhibited the number of tube formation in HUVECs and significantly suppressed the concentration and expression of VEGFA, indicating that ELF5 acted as an angiogenesis suppressor in RCC. Therefore, we confirmed that ELF5 exerted the anti-cancer effect in RCC.

DNA methylation is a key epigenetic regulatory mechanism in cancer development [27]. Tumor suppressor genes can be silenced by their promoter hypermethylation and can be activated again by demethylation [22]. Instead, oncogenes can be activated by hypomethylation [35]. The promoters of more than two hundred genes have been identified to be methylated in RCC. Lots of the frequently methylated genes in RCC have proven to affect cancer process, such as proliferation, apoptosis, invasion, and cell metabolism [36]. DNA methylation is established by DNMTs (DNMT1, DNMT3A, and DNMT3B). DNMT3A and DNM3B are indispensable to de novo DNA methylation [37]. DNMT1 retains DNA methylation pattern during cell division and can preferentially methylate hemimethylated DNA [38, 39]. Previous studies have confirmed that the downregulation of ELF5 expression is related to promoter methylation [40]. ELF5 downregulation in urothelial cancers is mediated by DNA methylation [25]. ELF5 is reported to be lowly expressed in luminal subtype breast cancer and its promoter methylation level is high [41]. We proved ELF5 was highly methylated in RCC cells and negatively correlated with DNMT1 and DNMT3B. DNMT1 and DNMT3B knockdown elevated ELF5 expression, however, ELF5 expression was notably rebounded after 5-Aza-dC treatment. Thus, we confirmed that DNMTs can promote ELF5 hypermethylation, leading to downregulation of ELF5 expression in RCC cells. The 5-Aza-dC treatment was also demonstrated to inhibit mouse tumor growth and upregulate ELF5 expression in tumor tissues, which showed the therapeutic potential in RCC by regulating ELF5.

Ubiquitination is a post-translational modification which exerts the vital function in assorted cellular processes [42]. Deubiquitination is an important opposing mechanism of ubiquitination [43]. Protein ubiquitination is not a unidirectional process, so it can be reversed by a type of protease called deubiquitinase (DUB) [44]. According to reports, approximately 100 DUBs are encoded by human genes and classified into six subclasses according to their Ub protease domains [45]. USP3 belongs to DUB and located on the human chromosome 15q22.3 [46]. The important function of USP3 in malignant tumors has been confirmed by some studies. For example, USP3 depletion promotes the development of colorectal cancer through decreasing SMAD4 expression [47]. USP3 can accelerate gastric cancer metastasis by enhancing the COL9A3/COL6A5 stability via deubiquitination [48]. It is reported that USP3-insufficient mice spontaneously develop tumors due to the destruction of chromosomal integrity [49]. The USP3-insufficient mice have a shorter lifespan and an increased risk of developing hematopoietic cancer [50]. Herein, we found USP3 was a target gene for ELF5 predicted by bioinformatics tools and USP3 was lowly expressed in KIRC samples. ELF5 was proved to combine with USP3 promoter, thereby transcriptionally activating USP3 expression in RCC cells. Subsequently, we found USP3 overexpression significantly suppressed cell malignant phenotypes and angiogenesis in RCC. This study proved USP3 can function as a tumor suppressor gene in RCC.

USP3 has been identified to have deubiquitination activity during cell activity in various malignant tumors [51]. For example, USP3 facilitates breast cancer cell growth via deubiquitinating KLF5 [52]. USP3 acts as a deubiquitinase of Aurora A to promote metastasis of esophageal squamous cell carcinoma [53]. Therefore, we further investigated the interaction protein of USP3 through bioinformatics analysis of BioGRID, HitPredict, MIST and Genemania databases. Among the screened proteins, we found WDTC1 was significantly downregulated in KIRC, indicating that WDTC1 may be an interacting protein of USP3 in renal cell carcinoma. Furthermore, we confirmed that USP3 and WDTC1 can interact endogenously and directly. WDTC1 expression was increased by USP3, while WDTC1 and USP3 expression can be increased by ELF5. Importantly, USP3 was confirmed to exert deubiquitination activity to inhibit the ubiquitination of WDTC1 in RCC cells. Therefore, we confirmed that ELF5 improved the stability of WDTC1 by driving USP3. WDTC1 is an anti-obesity gene and inhibits adipogenesis by the CRL4WDTC1 E3 ligase [54]. WDTC1 was initially identified in Drosophila, and its depletion could increase fat storage [55]. In colorectal cancer, WDTC1 can function as the oncogene to facilitate cancer development [56]. However, we discovered WDTC1 expressed at a low level in RCC cells, which was consistent with our bioinformatics predictions. Furthermore, WDTC1 upregulation significantly suppress RCC cell proliferation, migration, invasion, angiogenesis, and RCC tumor progression in vivo. WDTC1 depletion can reverse the inhibitory effects of ELF5 overexpression on RCC progression. These results demonstrated that ELF5 inhibited RCC development by upregulating WDTC1.

## Conclusion

Overall, this study proves that ELF5 is down-regulated expression in RCC due to DNA hypermethylation. ELF5 acts as a tumor suppressor though activating the transcription of USP3 to stabilizing WDTC1 in RCC. These findings may offer the new therapeutic targets for RCC.

### Electronic supplementary material

Below is the link to the electronic supplementary material.

Supplementary Material 1

## Data Availability

Original data generated in this study are available from the corresponding authors on reasonable requests.

## Abbreviations

5-Aza-dC: 5-Aza-2-deoxycytidine
BLCA: Bladder urothelial carcinoma
BRCA: Breast invasive carcinoma
CESC: Cervical squamous cell carcinoma and endocervical adenocarcinoma
CCK-8: Cell counting kit-8
ChIP: Chromatin immunoprecipitation
CHOL: Cholangio carcinoma
CHX: Cyclohexane
CM: Conditioned medium
COAD: Colon adenocarcinoma
Co-IP: Co-immunoprecipitation
DMEM: Dulbecco’s modified eagle medium
DNMTs: DNA methyltransferases
ELF5: E74-like transcription factor 5
ELISA: Enzyme linked immunosorbent assay
ESCA: Esophageal carcinoma
ETS: E26 transformation-specific
GBM: Glioblastoma multiforme
HNSC: Head and neck squamous cell carcinoma
HUVEC: Human umbilical vein endothelial cell
IHC: Immunohistochemistry
KICH: Kidney chromophobe
KIRC: Kidney renal clear cell carcinoma
KIRP: Kidney renal papillary cell carcinoma
LIHC: Liver hepatocellular carcinoma
LUAD: Lung adenocarcinoma
LUSC: Lung squamous cell carcinoma
MSP: Methylation-specific polymerase chain reaction
MVD: Microvessel density
NC: Negative control
PAAD: Pancreatic adenocarcinoma
PCPG: Pheochromocytoma and Paraganglioma
READ: Rectum adenocarcinoma
RCC: Renal cell carcinoma
RIPA: Radio immunoprecipitation assay
RT-qPCR: Reverse-transcription quantitative polymerase chain reaction
SARC: Sarcoma
SFM: Serum-free medium
SKCM: Skin Cutaneous Melanoma
STAD: Stomach adenocarcinoma
THCA: Thyroid carcinoma
THYM: Thymoma
UCEC: Uterine Corpus Endometrial Carcinoma
USP3: Ubiquitin-specific protease 3
VEGF: Vascular endothelial growth factor
WCL: Whole-cell lysates
WDTC1: WD40 and tetratricopeptide repeats 1
