# Supplementary material for: ELF5 drives angiogenesis suppression though stabilizing WDTC1 in renal cell carcinoma
Source: Mol Cancer. 2023 Nov 18;22:184. doi: 10.1186/s12943-023-01871-2 (PMC10656961; doi:10.1186/s12943-023-01871-2)
Supplement: Supplementary file 1 — Supplementary Material 1 [file 12943_2023_1871_MOESM1_ESM.docx]

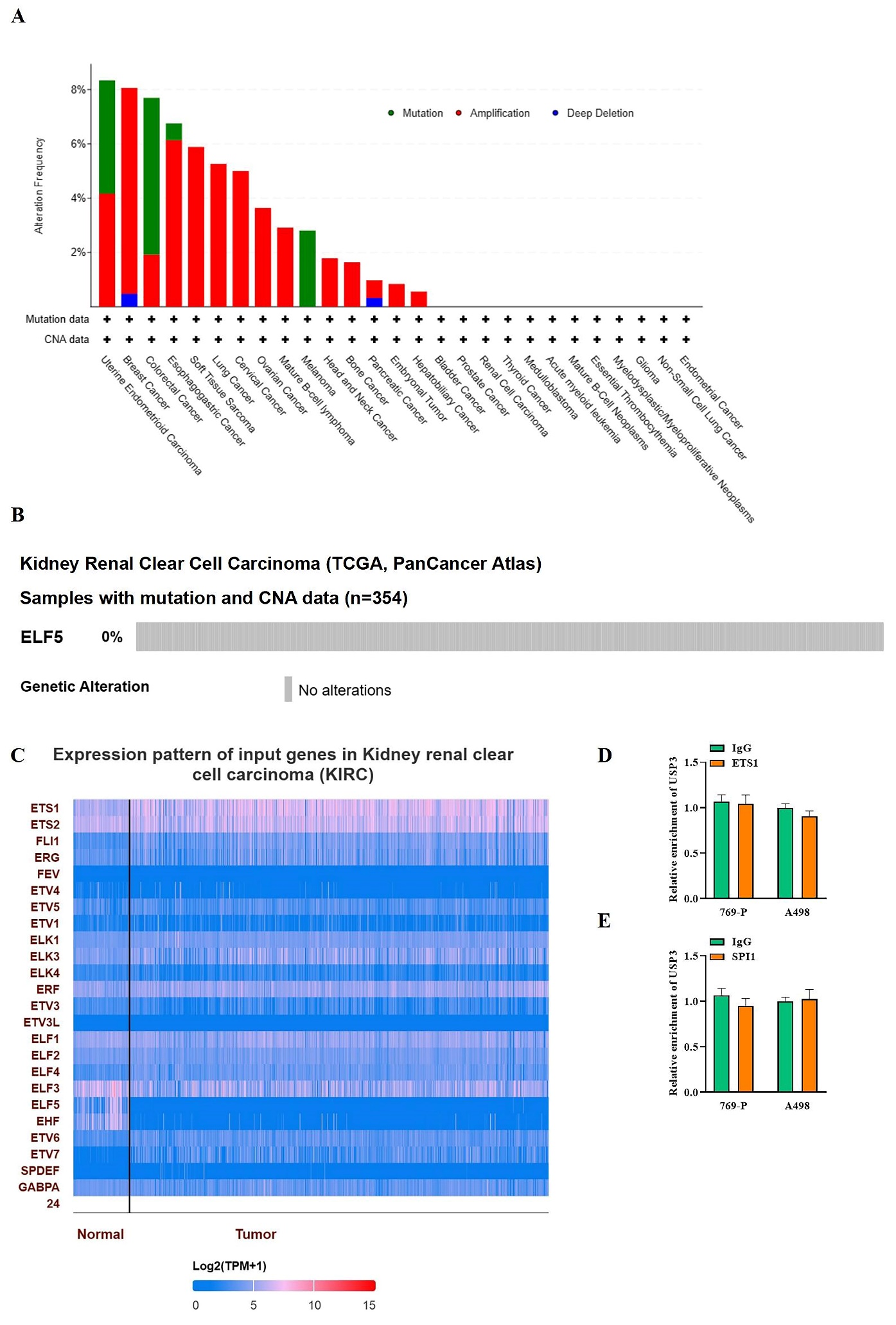


**Fig. S1 Expression pattern of** **ELF5 and ETS transcription factor family.**

(A) CNA and mutation frequency data of ELF5 in different cancer studies based on the cBioPortal platform (<http://www.cbioportal.org>). (B) Genetic alterations of ELF5 in Kidney Renal Clear Cell Carcinoma from the cBioPortal platform. (C) Expression pattern of members of the ETS transcription factor family. ChIP assays were performed to determine the binding relation between USP3 promoter and (D) ETS1 or (E) SPI1.


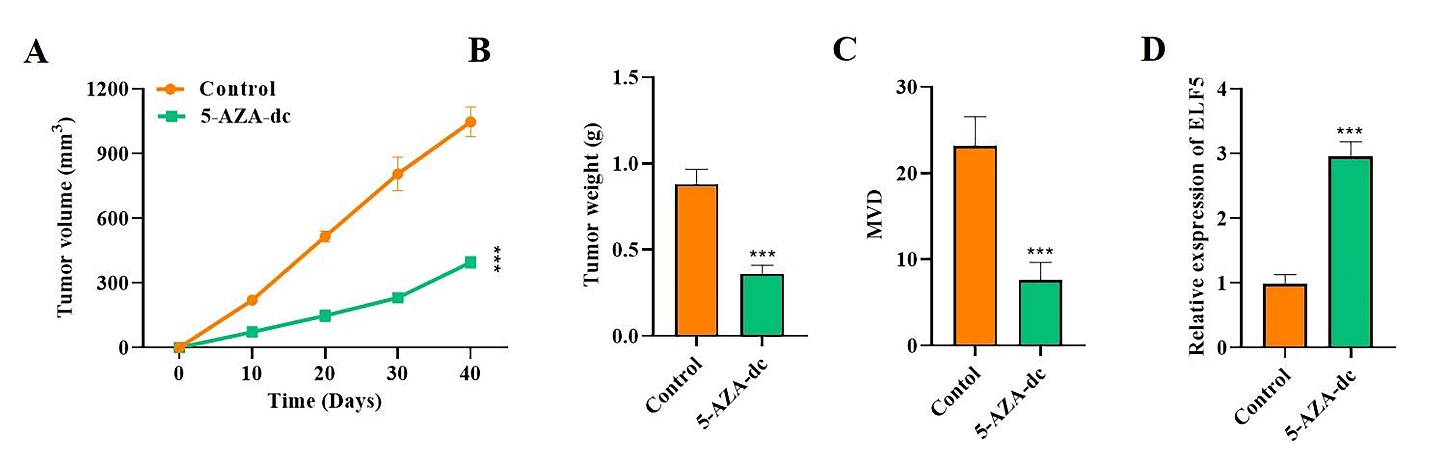


**Fig. S2 The effects of 5-AZA-dc on tumor growth.**

(A) Mouse tumor volume and (B) weight in the control and 5-AZA-dc groups. (C) MVD in tumor tissues of the control and 5-AZA-dc groups. (D) RT-qPCR analysis determined the ELF5 expression in tumor tissues. ^***^*p*<0.001.


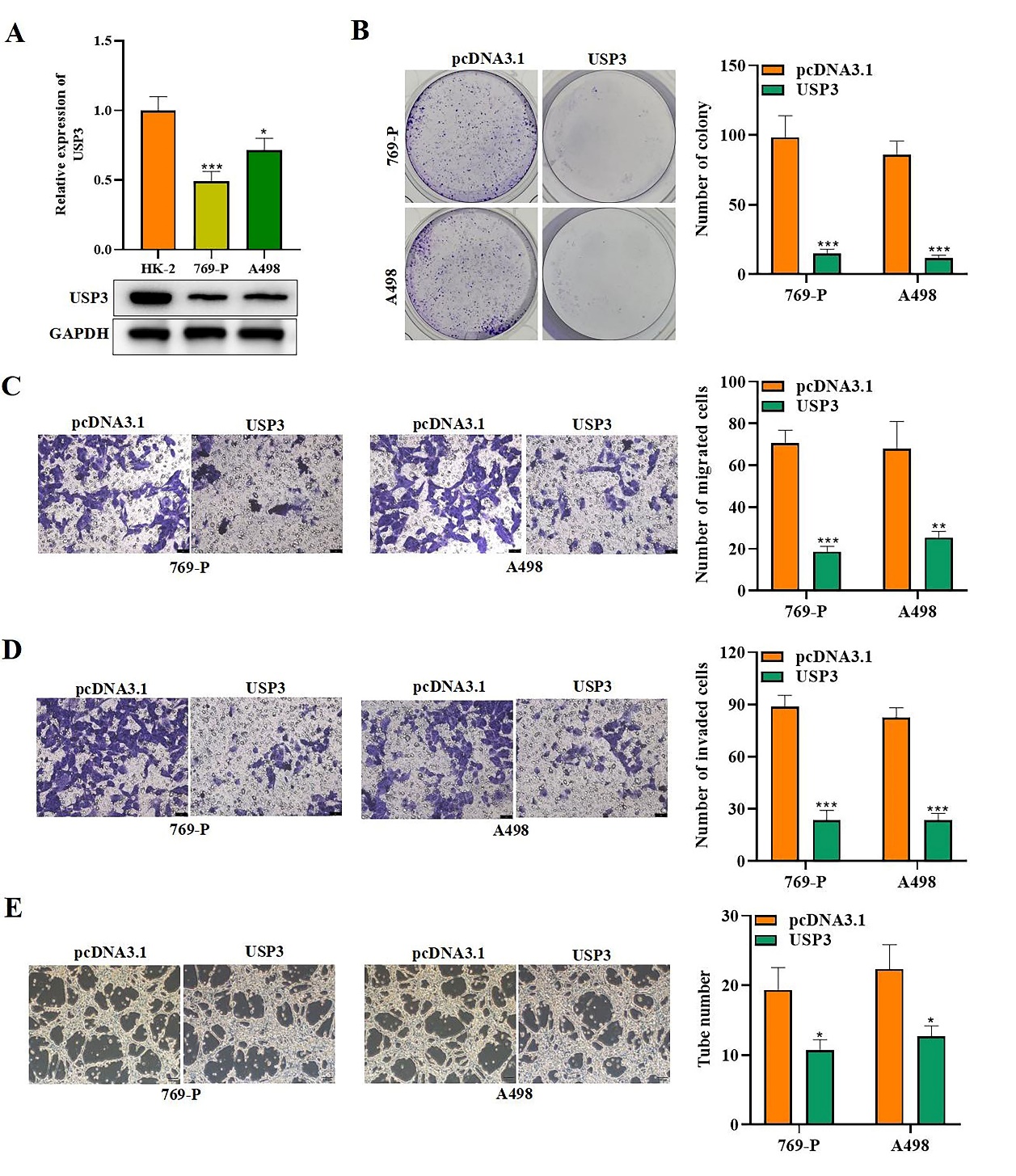


**Fig. S3 USP3 overexpression inhibited RCC cell malignant phenotypes.**

(A) RT-qPCR and western blot results of USP3 expression in HK-2, 769-P, and A498 cells. (B) Colony formation assay was employed for detecting cell proliferative capability when USP3 was overexpressed. (C-D) Transwell assay was carried out for testing cell migratory and invasive capabilities. (E) Tube formation assay was employed for testing angiogenesis of HUVECs stimulated by the conditioned medium collected from the USP3 overexpressed 769-P and A498 cells. ^*^*p*<0.05, ^**^*p*<0.01, ^***^*p*<0.001.
